# Snake Venom Disintegrin Inhibits the Activation of Toll-Like Receptors and Alleviates Sepsis through Integrin alphaVbeta3 Blockade

**DOI:** 10.1038/srep23387

**Published:** 2016-03-18

**Authors:** Chun-Chieh Hsu, Woei-Jer Chuang, Ching-Hu Chung, Chien-Hsin Chang, Hui-Chin Peng, Tur-Fu Huang

**Affiliations:** 1 Graduate Institute of Pharmacology, College of Medicine, National Taiwan University, Taipei, Taiwan; 2 Department of Biochemistry and Institute of Basic Medical Sciences, College of Medicine, National Cheng Kung University, Tainan, Taiwan; 3 Department of Medicine, Mackay Medical College, New Taipei City, Taiwan

## Abstract

Bacterial infection-induced sepsis is the leading cause of septic inflammatory disease. Rhodostomin (Rn), a snake venom disintegrin, was previously reported to interact with the αVβ3 integrin and the TLR4 on phagocyte in attenuating LPS-induced endotoxemia. In this report, we further evaluated the effects of Rn on TLR2-activated monocytes and its *in vivo* efficacy. Rn effectively suppressed the adhesion, migration, and cytokine release of Pam3CSK4-activated THP-1 cells. Rn specifically bound to integrin αVβ3 of TLR2-activated THP-1. Integrin αV and Akt siRNA transfection both restrained Pam3CSK4-elicited cytokine release. Rn decreased the Pam3CSK4-induced phosporylation of MAPKs, degradation of IκB and activation of FAK, Akt, c-Src and Syk. The Pam3CSK4-induced translocation of MyD88, a central adaptor of TLR2, to the cell membrane was also inhibited by Rn treatment. In the polymicrobial inflammatory caecal ligation and puncture model, Rn significantly reduced pro-inflammatory cytokine and chemokine release, alleviated tissue injury and elevated survival rate *in vivo*. Taken together, in addition to inhibiting the activation of TLR4, Rn exhibits anti-inflammatory activity through antagonizing the activation of phagocytes and interrupting the crosstalk between αVβ3 and TLR2-dependent signaling pathways.

The innate immune system is the first line to identify and defense microbial pathogens protecting the host from infection^1^. In human, at least ten toll-like receptors (TLRs) specifically recognize different microbial patterns to initiate signaling pathways leading to inflammation^2^. TLR4 responses for recognition of a major component from Gram-negative bacteria, lipopolysaccharides (LPS), while TLR2 forms heterodimer with either TLR1 or TLR6 to recognize several pathogen-associated molecular patterns, including peptidoglycan from Gram-positive bacteria. LPS, an outer membrane component of Gram-negative bacteria, is a well-characterized microbial antigen in immunology^3^. After TLR4 activation by LPS, the activation of mitogen-activated protein kinase (MAPK) signaling pathway and transcription factor, nuclear factor κB (NFκB) are triggered in phagocytes^3^.

Integrin is another important receptor family of immune cells. β2 and β3 integrin forms heterodimer with α subunit to regulate leukocyte trafficking and function^4^,^5^. Bacterial interaction with eukaryotic cells also involves the engagement of integrins. Among them, integrin αVβ3 promotes the internalization of bacteria, including *Borrelia burgdorferi, Neisseria spp., Porphyromonas gingivalis and Streptococcus spp*.^6^. Additionally, though activating mechanisms of TLR2 are not well understood, a role of vitronectin and αVβ3 in initiating TLR2 responses to bacterial lipopeptides was revealed^7^. Integrin αVβ3 was also reported to serve as coreceptor of TLR2 in epithelial cells^8^. Therefore, β3 integrin may play important role during TLR2 agonist-induced activation, thus providing an additional target for drug intervention.

We previously reported that a disintegrin, rhodostomin (Rn), interrupts the LPS-induced activation of phagocytes through interaction with αVβ3 integrin of monocytes/macrophages, contributing to its anti-inflammatory protection in LPS- induced endotoxemia *in vivo*^9^. Disintegrin, an RGD-containing peptide family, binds with high affinity to integrins expressed on platelets, vascular endothelial cells and some tumor cells^10^. Rn, purified from *Calloselasma rhodostoma* snake venom, comprises 71 amino acids containing RGD domain^11^. We reported that Rn interrupts the interaction between fibrinogen and αIIbβ3 and attenuates platelet aggregation^11^,^12^. We also demonstrated that Rn exhibits antiangiogenic activity *in vivo* and *in vitro* through endothelial αVβ3 blockade^13^.

In this report, we used recombinant Rn^14^ to explore the anti-inflammatory effect of disintegrin on Pam3CSK4 (a TLR2-specific agonist)-induced activation of phagocytes and the mechanism involved. Furthermore, its *in vivo* anti-inflammatory efficacy in a polybacterial infection caecal ligation and puncture (CLP)-model was examined.

## Results

### Rn inhibits Pam3CSK4-stimulated cytokine production *in vitro*

**Figure 1:**
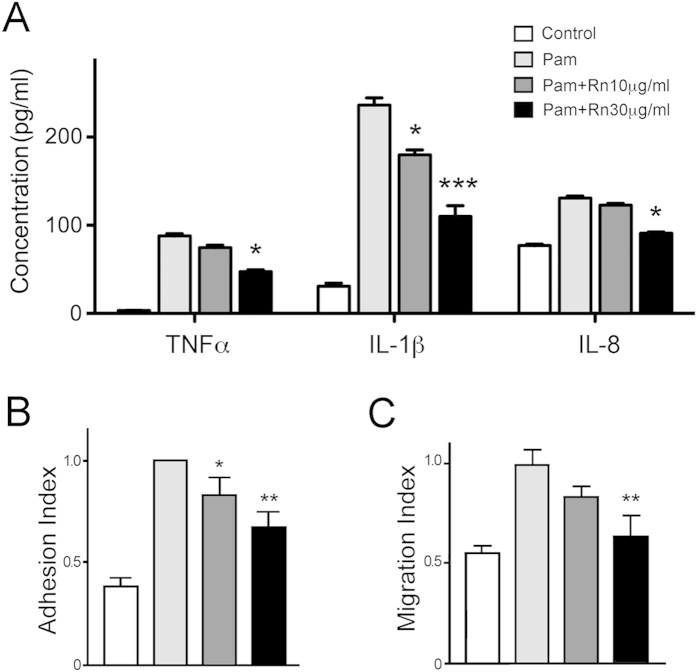
Effects of Rn on cytokine production, cell adherence and migration of TLR2-activated phagocytes. (**A**) THP-1 cells were treated with different concentrations of Rn after cell incubation in the presence (Pam) or absence (Control) of Pam3CSK4. After 24 hr, the supernatant of the culture were collected and concentrations of cytokines, TNFα, IL-1β and IL-8, were measured by ELISA. (**B**) TLR2-activated THP-1 cells were treated with increasing concentrations of Rn and incubated. The adhesion index was calculated as fold of adhered cells normalized to that of only Pam3CSK4-treated cells. (**C**) TLR2-activated THP-1 cells were treated with Rn and migrated through gelatin-coated transwells. The migration index was calculated as fold change of migrated cells normalized to that of only Pam3CSK4-treated cells. Control, non-activated control. Values are the mean ± S.E. of three independent experiments, and *p < 0.05, **p < 0.01, ***p < 0.001 compared with only Pam3CSK4-activated groups.

The concentrations of cytokines in THP-1 media were analyzed by ELISA (Fig. 1A). Incubation of THP-1 with Pam3CSK4 alone raised the concentration of cytokines, including IL-1β, IL-8 and TNFα (236.03 ± 8.39, 130.64 ± 2.51 and 87.86 ± 2.61 pg/ml), compared to control group (30.67 ± 3.58, 76.80 ± 1.85 and 3.32 ± 0.12 pg/ml). However, as compared to those of Pam3CSK4 only group, Rn (30 μg/ml, 3.86 μM) significantly decreased these cytokines levels (IL-1β, 109.94 ± 12.27; IL-8, 90.99 ± 1.20 and TNFα, 47.22 ± 2.26 pg/ml, respectively).

### Rn blocks the adhesion and migration activity of monocytes

Leukocytes attach to and penetrate throughout the blood vessel prevalently in inflammatory condition^4^. We used Pam3CSK4 to induce inflammatory activities of monocytic cell line, THP-1, through TLR2 binding. Pam3CSK4 is a potent activator of TLR2, which is mediated by the interaction between TLR2 and TLR1^15^. THP-1 were seeded in the presence of serum, and TLR2-activated cell adherence was assayed (Fig. 1B). THP-1 cell adhesion was significantly enhanced by Pam3CSK4 stimulation, but this increase was inhibited by Rn treatment in a concentration-dependent way. We also coated transwells with gelatin to examine the influence of Rn on TLR2- induced THP-1 transmigration (Fig. 1C). The Pam3CSK4-induced increase of migrated-cell number was also inhibited by Rn. These results indicate that Rn is an effective inhibitor on TLR2-mediated THP-1 adherence and transmigration through extracellular matrix.

### Rn specifically binds to αVβ3 integrin to affect TLR2-activated THP-1 cell adherence and cytokine production

**Figure 2:**
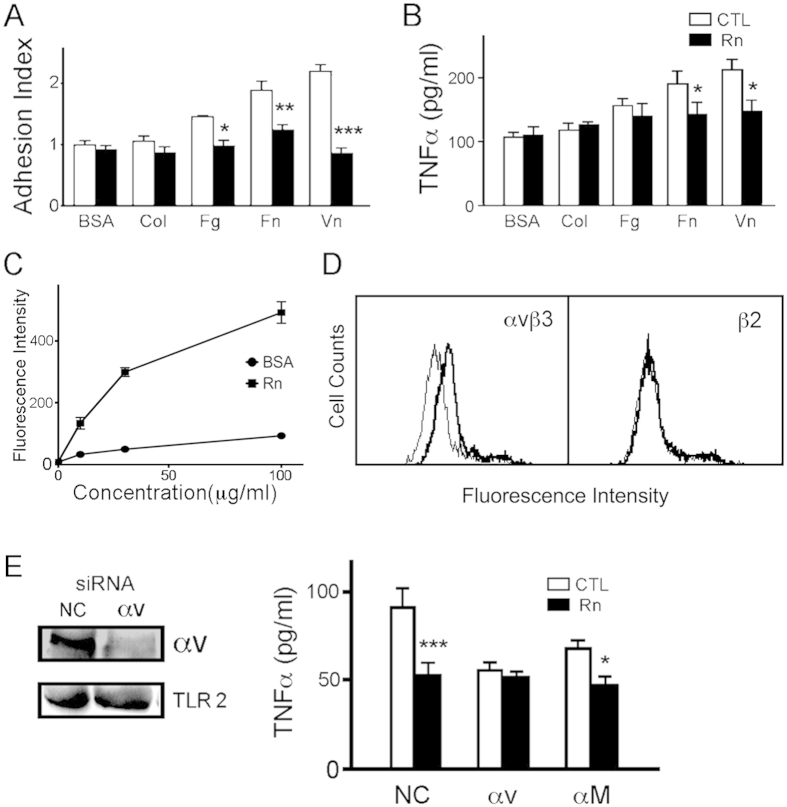
Rn inhibits the adhesion, cytokine release of the TLR2-stimulated THP-1 through blockade of integrin αVβ3. (**A**) Adhesion and (**B**) TNFα production by Pam3CSK4-stimulated THP-1 cells seeded on plates coated with albumin (BSA), collagen (Col), fibrinogen (Fg), fibronectin (Fn) and vitronectin (Vn), in the absence (CTL) or presence of Rn (Rn, 30 μg/ml). (**C**) Pam3CSK4-activated THP-1 cells were incubated with increasing concentration of FITC-Rn before the fluorescence intensity measurement by flow cytometry. Total binding (square) and non-specific binding (circle) were determined by FITC-Rn and FITC-BSA, respectively. (**D**) Pam3CSK4-activated THP-1 cells were incubated with Rn (30 μg/ml, bold line) or not (thin line) before probing with anti-αVβ3 or anti-β2 mAbs, respectively. (**E**) THP1 cells were transfected with integrin αV (αV), integrin αM (αM) or negative control (NC) siRNA for 48 hr followed by different assays. Western blotting for integrin αV and TLR2 (left panel). TNFα release measured with the ELISA kit (right panel). Blank bar, untreated control (CTL). Black bar, incubated with Rn (Rn). Means ± SEM of three separate experiments were checked for statistical difference. *p < 0.05, **p < 0.01, ***p < 0.001, compared with Pam3CSK4-activated control groups (CTL).

We previously showed that Rn specifically binds to integrin αVβ3 on TLR4-activated phagocytes^9^. To ask the functional roles of various integrin in TLR2 signaling, Pam3CSK4-pretreated THP-1 cells were applied into wells precoated with matrix, including bovine serum albumin (BSA), collagen, fibrinogen, fibronectin and vitronectin. The adhesion (Fig. 2A) and TNFα release (Fig. 2B) of monocytes in response to Pam3CSK4 were raised, and especially fibronectin and vitronectin were used as matrices. Rn efficiently blocked the enhanced adhesion and TNFα release to the basal level, implying the involvement of integrin αVβ3.

The interaction between Rn and monocytes was assayed by flow cytometry. The total bound fluorescence intensity of FITC-Rn to Pam3CSK4-activated THP-1 was augmented as concentration increased, whereas FITC-conjugated BSA showed a very low binding activity (Fig. 2C). We also detected that Rn selectively reduced the binding fluorescence intensity of anti-αVβ3 mAb, with little effect on anti-β2 mAb binding (Fig. 2D). These data confirmed that αVβ3 is the the specific binding site of Rn on Pam3CSK4-stimulated THP-1 cells.

We then tested the involvement of integrin αV in the TLR2-stimulated THP-1 by using a combination of two siRNA duplexes specific for integrin αV. Endogenous expression of integrin αV was reduced after transfecting with integrin αV siRNA in contrast to a negative control siRNA. TLR2 expression was not affected by transfecting with negative control or αV siRNA, displaying a specificity of integrin αV siRNA (Fig. 2E). The cell viability showed no significant difference between cells transfected with αV and negative control siRNA (data not shown).

Upon stimulation by Pam3CSK4, αV siRNA-transfected THP-1 cells exhibited reduced TNFα release as compared to negative control, whereas Rn failed to further inhibit TNFα release of the αV siRNA-transfected cells (Fig. 2E, right panel). Transfection of αM siRNA also effectively decreased Pam3CSK4-elicited cytokine production, however, Rn still showed a further inhibition on αM siRNA-transfected cells (Fig. 2E). These data reveal that Rn interacts with integrin αV, but not with integrin αM, on THP-1 cells.

### Rn inhibits TLR2 signaling pathway

**Figure 3:**
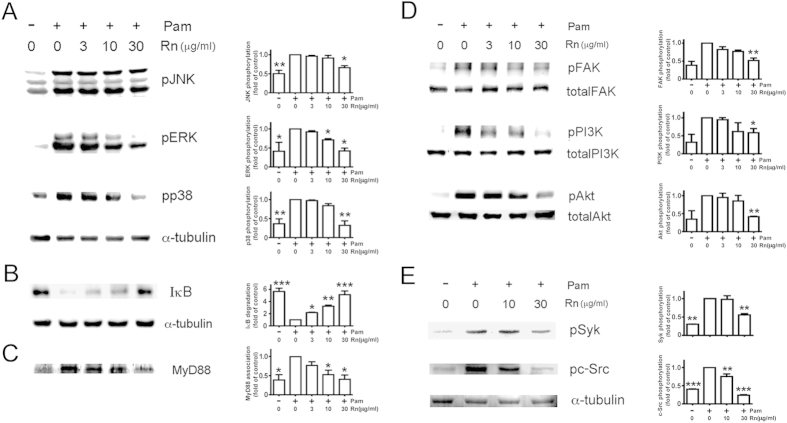
The effect of Rn on TLR2-induced activation of signaling pathways in THP-1 cells. Pam3CSK4-activated THP-1 cells were incubated with different concentrations of Rn, and cell lysates and cell membrane fraction were analyzed with Western blotting assay. Immunoblotting analyses of level of (**A**) Phosphorylated p38, JNK and ERK, (**B**) IκB in THP-1 cell lysate and (**C**) MyD88 in THP-1 cell membrane fraction. (**D**) Phosphorylated and total FAK, PI3K and Akt and (**E**) Phosphorylated c- Src and Syk and α-tubulin were examined. The pattern is one example of three independent experiments with similar results. Means ± SEM of three separate experiments were checked for statistical difference. *p < 0.05, **p < 0.01 and **p < 0.001.

Previous study indicates that MAPKs and NFκB signaling pathway participate in the TLR2-induced production of cytokines and play important roles in phagocytes^16^. After Pam3CSK4 (1 μg/ml) treatment, ERK, JNK, and p38 were activated obviously in THP-1 cells (Fig. 3A). As THP-1 cells were pretreated with various concentration of Rn and assayed by Western-blotting, Rn (30 μg/ml) significantly decreased the Pam3CSK4-stimulated activation of JNK, ERK and p38 (Fig. 3A). Meanwhile, Rn also reversed Pam3CSK3-induced IκB degradation in a concentration-dependent way (Fig. 3B). These data indicate that the inhibition of Rn on cytokine release is due to the blockade of MAPKs activation and IκB degradation.

Nevertheless, TLR2 signaling pathway consists of a MyD88-dependent upstream pathway and MyD88 associates to TIR domain on TLRs, resulting in the activation of MAPK and NFκB^2^. As shown in Fig. 3C, in the absence of stimulation, only little MyD88 in membrane fraction was detected, however, an increased immunoreactivity of membranous MyD88 was observed 15 min following TLR2 stimulation. Moreover, Pam3CSK4-induced TLR2 associated MyD88 was concentration-dependently inhibited by Rn pretreatment (Fig. 3C).

### Rn inhibits integrin-related signaling pathway

Focal adhesion kinase (FAK) is involved in integrin-dependent signal transduction and tyrosine 397-phosphorylation is the recognition of FAK activation^17^,^18^. Rn (30 μg/ml) significantly reversed FAK phosphorylation upon Pam3CSK4 stimulation (Fig. 3D). Moreover, Pam3CSK4-activated phosphorylation of FAK-downstream protein kinases, PI3K and Akt, were also attenuated by Rn treatment.

Previous work has revealed that Syk is important for neutrophil integrin signaling^19^. Src-family kinases (SFKs) also are critical for the initiation of integrin signaling in macrophage^20^. As TLR2 was activated upon incubation with Pam3CSK4, Syk and c-Src phosphorylation significantly increased (Fig. 3E). Rn (30 μg/ml) also inhibited Syk and c-Src phosphorylation in response to Pam3CSK4 stimulation (Fig. 3E).

### Integrin αV-TLR2 complex in monocytes

**Figure 4:**
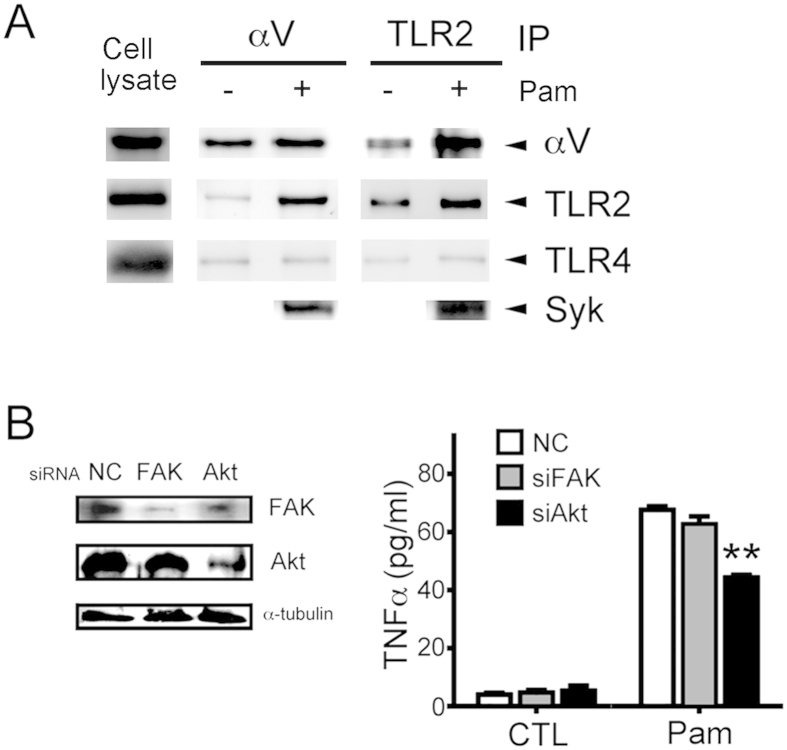
Integrin β3 forms a complex with TLR2 in THP-1 cells. (**A**) Immunoassay of THP-1 cells stimulated with Pam3CSK4 (Pam, 1 μg/ml) for 5 min or not, then lysed and immunoprecipitated (IP) with anti-integrin αV or anti-TLR2 and analyzed by immunoblot with anti-integrin αV, TLR2, TLR4 or Syk mAbs. Cell lysis control was shown in left panel. The pattern is one example of three independent experiments with similar results. (**B**) THP-1 cells were transfected with FAK, Akt or negative control (NC) siRNA for 48 hr followed by Pam3CSK4 (Pam) treatment, and TNFα release measured with the ELISA kit. Means ± SEM of three separate experiments were checked for statistical difference. **p < 0.01, compared with negative control groups (NC).

To investigate the direct interaction between integrin αV and TLR2, we examined this complex formation by co-immunoprecipitation assays in Pam3CSK4-activated THP-1 cell lysates (Fig. 4A). We found that integrin αV was significantly precipitated along with TLR2, but not TLR4, in TLR2-activated THP-1 cells. It was also found that Syk was also incorporated in the complex.

### The effect of integrin signaling pathway on TLR2-activated monocytes

We further investigated the role of integrin-signaling pathway molecules on TLR2- dependent activation of monocytes. As stimulated by Pam3CSK4, Akt siRNA-transfected THP-1 cells exhibited a reduced TNFα release as compared to negative control (Fig. 4B). However, FAK siRNA-transfection had no effect on TNFα release as compared to negative control.

### Effect of Rn on CLP-induced cytokine production, plasma glucose concentration, mean blood pressure changes and mortality in mice

**Figure 5:**
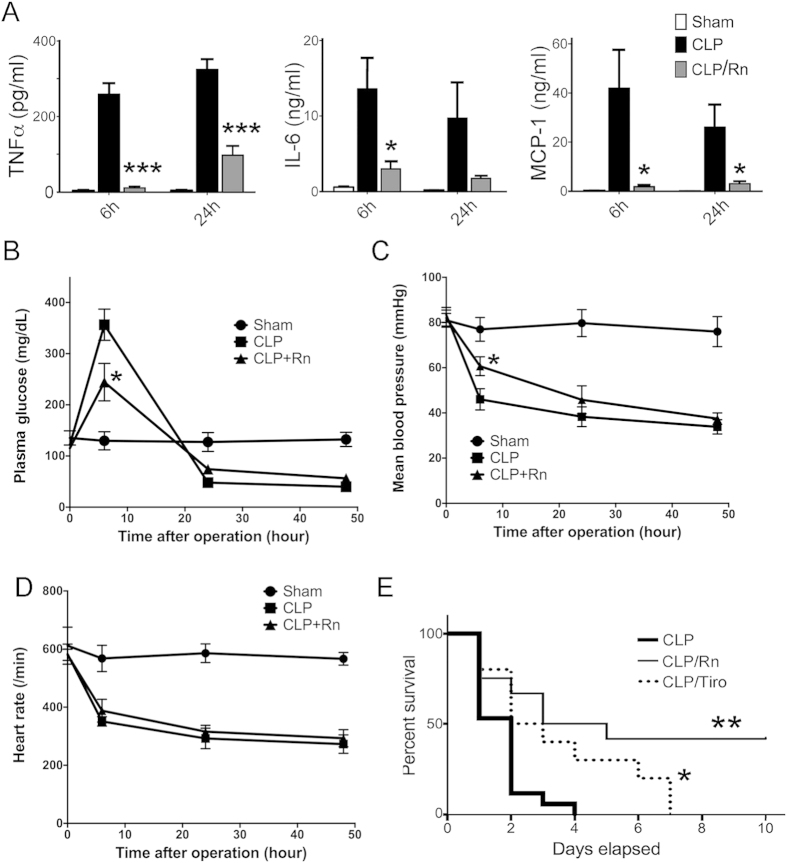
The protective effects of Rn on cytokine production and mortality in CLP model. (**A**) Mice were anesthetized and blood was collected for plasma isolation, and concentration of cytokines was measured by ELISA. Changes in plasma glucose levels (**B**), Mean blood pressure (**C**,**D**) Heart rate at 6, 24 and 48 hr after sham operation (circle), CLP (square) and CLP with Rn (triangle) were recorded (N = 5–8). (**E**) Mice (n = 15) were administered intravenously, with Rn (10 mg/kg) or tirofiban (Tiro, 0.1 mg/kg) immediately after CLP. Animal survival was monitored everyday after CLP for 10 days. Control CLP mice were administered with sterile saline (n = 10). A Log-Rank (Mantel-Cox) survival analysis was used for determination of overall survival rates vs. CLP-treated mice. *p < 0.05, **p < 0.01 vs. CLP.

Among the experimental sepsis models, host immune responses in caecal ligation and puncture (CLP) model most mimic the course in human disease, including haemodynamic and metabolic phases of human sepsis^21^. We carried out the CLP model to examine the effect of Rn on this complex infection animal model. Since excessive elevation of pro-inflammatory cytokines in circulation is a major contributor to remote organ injury after CLP, we examined the plasma levels of pro-inflammatory cytokines. It was observed that the concentrations of cytokines were low or undetectable in sham mice (data not shown). CLP-mice exhibited elevated cytokines levels at 6 h and 24 h, including TNF-α, IL-6 and MCP-1. As Rn (10 mg/kg) was intravenously administered following CLP instantly, the increases of these cytokines were profoundly inhibited (Fig. 5A).

In the sham group, plasma glucose levels were not altered during the experiment period (Fig. 5B). CLP induced biphasic changes in plasma glucose, displaying hyperglycemia at 6 hr after CLP and followed by hypoglycemia from 24 hr onwards. Pretreatment of CLP mice with Rn significantly reduced hyperglycemia at 6 hr after CLP as compared with CLP group (p = 0.0432), but had no significant effect on hypoglycemia after 24 hr (Fig. 5B). Mean blood pressure and heart rate were not affected in sham group during the experiment period, but hypotension and bradycardia occurred after CLP (Fig. 5C,D). However, Rn pretreatment improved hypotension at 6 hr after CLP (p = 0.0418) (Fig. 5C).

We first performed a 10-day survival study to confirm the effect of Rn on CLP-induced polymicrobial sepsis. CLP mice group showed 100% mortality on day 4, whereas Rn-treated mice group showed 41.67% mortality on day 10 (n = 12) (Fig. 5E). The mean survival time of the control septic mice was about 1.7 days, whereas that of Rn-treated septic mice was prolonged to about 5.5 days (more than 10 days was counted as 10 days). Therefore, Rn-treatment exerted a protective effect as shown by prolonging survival rate of severely septic mice. We also assayed the effect of a disintegrin-like αIIbβ3 antagonist, tirofiban, on CLP mice, which showed 100% mortality on day 7 (n = 10) and the mean survival time about 3.5 days (Fig. 5E).

Taken together, Rn treatment efficiently lowers the plasma pro-inflammatory cytokines and mortality in septic mice. Moreover, administration of tirofiban, an anti-platelet disintegrin-like drug, also improved the survival rate of CLP mice. However, Rn displayed a better efficacy than tirofiban.

### Rn alleviates CLP-induced tissue injury as examined by histochemistry

**Figure 6:**
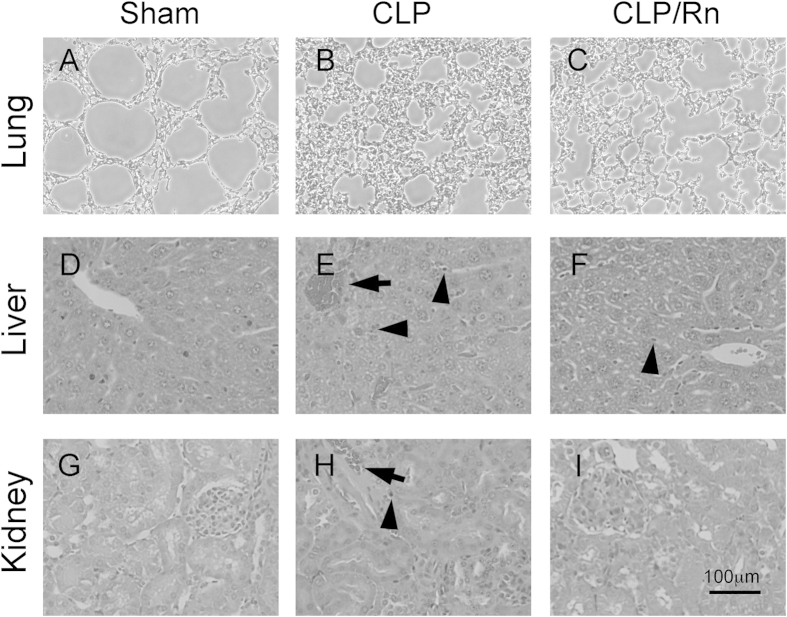
The effects of Rn on tissue inflammation in CLP-induced sepsis model *in vivo*. Morphological changes in mouse lung (**A–C**), liver (**D–F**) and kidney (**G–I**) sections of BAL/c mice on CLP-induced sepsis (H&E, original magnification, ×200). (**A**,**D**,**G**; Sham) Sham control mice, (**B**,**E**,**H**; CLP) CLP-induction mice, (**C,F,I**; CLP/Rn) CLP-induction mice treated with Rn. Infiltrated leukocytes (arrowhead) and occlusive vessels (arrow) in in liver and kidney were indicated.

In comparison with sham mice (Fig. 6A,D,G), histological examination of CLP-induction mice (Fig. 6B,E,H) showed that lung was characterized by the presence of leukocytes in the interstitium and alveoli, and thickening of the alveolar wall (Fig. 6B), while liver section exhibited leukocyte infiltration in parenchyma and showed peri-vascular infiltration (Fig. 6E). The kidney was also damaged by CLP induction with evidenced glomerular hypercellularity (Fig. 6H). We also found some occlusive vessels in liver and kidney (Fig. 6E,H). However, the treatment of Rn (Fig. 6C,F,I) protected mice from these organ damage caused by CLP.

## Discussion

In this report, we observed that a snake venom disintegrin, Rn, suppresses the inflammatory responses of TLR2-stimulated monocytes. The binding of Rn towards THP-1 monocyte shows a saturated manner as its concentration increases, indicating that the interaction between Rn and monocyte is receptor-mediated. Furthermore, flow cytometry assay also showed that Rn selectively inhibited the binding of an αVβ3-specific mAb to THP-1 (Fig. 2). We revealed that the binding with vitronectin or fibronectin led to the increase of Pam3CSK4-elicited monocyte adherence and cytokine production; however, silencing or Rn-blockage of integrin αV attenuated these enhanced activities (Fig. 2). It is concluded that the anti-inflammatory effect of Rn depends on its blockage of the interaction between integrin αVβ3 and ligands.

Integrin αVβ3 is an important receptor of the RGD-containing ligands, including fibrinogen, vitronectin, osteopontin and disintegrins^7^. Integrins αVβ3 and α4β1 acts as the binding receptor of monocytic cells to some pro-inflammatory ligands, like monomeric C-reactive protein, fractalkine and matrices^7^,^22^,^23^. TNFα production from phorbol myristate acetate (PMA) activated THP-1 cells is also integrin αVβ3 and adhesion-dependent^24^. The antagonists of these integrins exert the anti-inflammatory actions through blocking ligands-integrin interaction. The effect of Rn on THP-1 in our data also indicates that integrin αVβ3 coordinated TLR2 responses to Pam3CSK4. Substantial literatures also support the theory that αVβ3-integrin plays a considerable role in the behavior of phagocyte and osteoclast^25^. These data indicate that integrin αVβ3 coordinates microbe-induced cytokine release and phagocyte-stimulated inflammation, and provide a potential target for clinical medication.

The signaling pathway of some TLRs, including TLR-2, -4, and -9, are regulated by MAPKs, responding to extracellular stimulation, regulating cellular activities and mediating cytokine and chemokine release^26^. Rn inhibits the Pam3CSK4 enhanced phosphorylation of MAPKs, resulting in a significant attenuation of cytokine expression at a transcription level. Besides activating MAPK pathways, Pam3CSK4 can also activate integrin signaling, e.g., phosphorylations of FAK signaling pathway. In TLR2-stimulated monocytes, we found that Rn inhibited not only phosphorylation of FAK/PI3K/Akt, but also phosphorylation of c-Src/Syk (Fig. 4). It implies that Rn blocks TLR2-activated integrin signaling to decrease phosphorylation of MAPKs.

**Figure 7:**
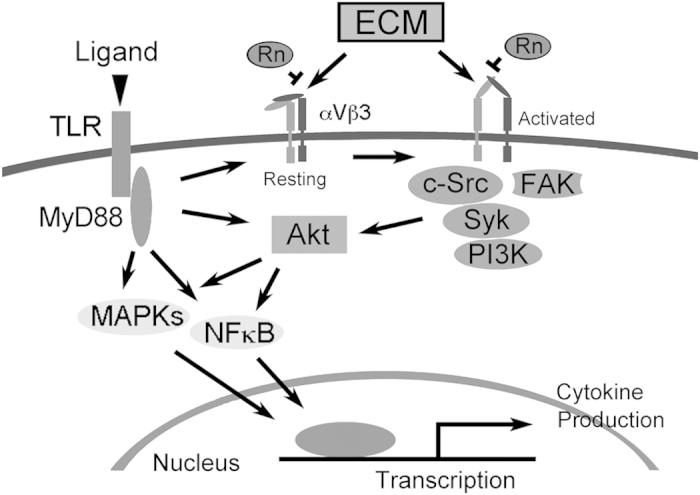
The hypothetical anti-inflammatory mechanisms of Rn in phagocyte. After being stimulated by ligand (i.e. Gram-positive or -negative bacteria), TLR activates the MyD88-dependent pathway through the MyD88-IRAK-TRAF6 complex, which in turn activates the downstream MAPKs and NFκB activation. In addition, integrin αVβ3 was also activated by inside-out signaling from the ligation of TLR, thus triggers downstream c-Src/Syk and FAK activation. Ligands (extracellular-matrix, ECM; vitronectin, fibronectin, etc.) binding to integrin αVβ3 also facilitates downstream signaling pathway. Rn possesses anti-inflammatory effects mainly through blocking the αVβ3 induced NFκB and MAPK pathways, and MyD88-dependent TLR signaling pathway in production of cytokines in phagocytes. Taken together, Rn suppresses release of inflammation-associated mediators (cytokines), inhibits cell adhesion and migration *in vitro* and even attenuates the acute inflammation of mice caused by bacterial infection in mice.

To further clarify the downstream signaling pathway crosstalk of TLR2 and integrin αVβ3, we considered FAK and Akt, two typical molecules activated in the integrin signaling pathway^19^. Though Pam3CSK4 activated FAK (Fig. 3D), our data revealed that FAK siRNA transfection has no effect on TLR2-dependent cytokine release (Fig. 4B). Besides being the downstream of integrin, Akt also involves in several signaling pathways^19^,^27^,^28^. Binding of TLRs turns on intracellular signal-transduction pathways, which leading to activate transcriptional factors such as interferon regulator factors (IRFs), PI3K/Akt, AP-1, and NFκB. In response to viral and bacterial components, Akt serves as a hub in the αVβ3-integrin-promoted TLR2 signaling^8^. We also observed that Akt siRNA transfection reduced TLR2-dependent cytokine release from Pam3CSK4-stimulated monocytes (Fig. 4B). Akt plays a key role in the cross-talk route between integrin αVβ3 and TLR2 (Fig. 7).

The inflammatory phenotype in *motheaten* mice primarily results from dysregulated neutrophil integrin signal transduction due to increased SFK and Syk activity, but deletion of Syk or SFK in neutrophils would reverse the inflammatory disease^29^. Src, together with Syk, are also important molecules activated in the integrin signaling pathway^16^. The activation of TLR2 also stimulated the phosporylation of Src and Syk and Rn concentration-dependently attenuated these activation (Fig. 3E). TLR2 forms complex with integrin αVβ3 in stimulated-monocytes, and Syk also can be found in the complex (Fig. 4A). From these results, it is suggested that Rn attenuates TLR2 signaling through αVβ3 and c-Src/Syk-dependent signaling pathway as illustrated in Fig. 7.

It is critical that innate immune system senses the microbes and turns on the immune responses. TLRs cooperate with other microbe-identification protein to take efficient responses against pathogens^2^, but chronic or excessive response may injure tissue and organ^30^. For instance, TLR2 and TLR4 participate in the pathogenesis of atherosclerosis, autoimmune colitis, systemic lupus erythematosus (SLE), diabetes and Alzheimer’s disease^31^. Although several TLR blockers presently are under ongoing clinical trials, but none was accepted for clinic medication^32^,^33^. Thus, inhibition of immoderate TLR activation is a therapeutic target being enthusiastically investigated on these sickness^34^,^35^. The potential application of Rn as an inhibitor of TLR-2 and TLR-4 activation provides a promising lead for drug development in infectious diseases induced by complicated microbial patterns.

Sepsis is caused by systemic reactions to severe inflammation. The leading cause of sepsis is bacterial infections, including both Gram-positive and Gram-negative bacteria. Though sepsis and septic shock cause million death annually worldwide, the only antisepsis agent, Xigris (human recombinant activated protein C/Drotrecogin alfa), was withdrawn from the market after a follow-up placebo-controlled trial due to lack of beneficial effect on mortality^36^. Polymicrobial sepsis elicited by CLP is the most often utilized model for researching complex inflammatory pathology and pharmacology^29^. Since Rn is an inhibitor of both TLR-2 and -4 signaling, it effectively attenuated LPS-endotoxemia syndromes^9^ and reduced mortality in CLP model. Our data showed that the crosstalk between integrin αVβ3 and TLRs signaling pathways is important and worthy to be further investigated.

Activation of the coagulation system leading to microvascular thrombi is the cause of multiple organ failure, and correlates with mortality in severe sepsis^3^. In LPS-induced endotoxemia model, a moderate inhibitory effect of heparin on cytokine release was also observed^9^. Tirofiban, a specific αIIbβ3 antagonist, also showed a significant, but less-pronounced beneficial effect on the survival rate of septic mice (Fig. 5B). These anti-coagulation or anti-platelet drugs may improve organ failure in the CLP-challenged host. Rn effectively blocks platelet aggregation and adherence to various matrices, preventing platelet activation and thrombus formation. Therefore, we would not exclude the possible contribution of its anti-thrombotic activity through platelet αIIbβ3 antagonism *in vivo*.

Rn markedly blocked phagocyte activity and reduced cytokine production of THP-1 cells stimulated by both Gram positive and negative bacteria, indicating that integrin αVβ3 on phagocytes not only promotes cell adherence but also assists the innate inflammatory activities induced by TLR2. Moreover, the blockade of integrin αVβ3 effectively protects the animal afflicted by complex bacterial-infected inflammatory diseases. However, the detail mechanism regarding how Rn interferes with the TLRs-elicited signaling is under exploration.

## Materials and Methods

### Materials

Enzyme-linked immunosorbent assays (ELISA) kit for IL-1β, IL-6, IL-8 and TNFα were purchased from BioLegend (San Diego, USA). Anti-phosphotyrosine mAbs, including anti-phosphorlated p38, ERK, JNK, FAK, PI3K, Akt, c-Src and Syk and IκB, MyD88 and anti-α-tubulin and FITC conjugated anti-mouse IgG were purchased from Santa Cruz (CA, USA). Antibodies against human integrins, αV (P3G8), β2 (MEM48) and αVβ3 (LM609), were purchased from Millipore (Temecula, CA, USA). Antibodies against human TLR2 and TLR4 were purchased from Santa Cruz (CA, USA). BCECF/AM was from Molecular Probes (Leiden, The Netherlands). RNAi-Mate Transfection Reagent were purchased from MDBio, Inc. (Taiwan). Gelatin and other chemicals were from Sigma Chemicals, Co. (St Louis, Mo, USA). Recombinant rhodostomin (Rn) was kindly provided by Dr. WJ Chuang^14^.

### Cell culture

The human monocyte, THP-1, was acquired from the American Type Culture Collection and was grown in RPMI-1640 media with 10% FBS. The cell was maintained at 37 ^o^C in an atmosphere containing 5% CO_2_.

### Cytokine production by TLR2-activated phagocytes

After monocytes were incubated with Pam3CSK4 (1 μg/ml) and various concentrations of Rn for 24 h, media were collected by centrifugation. The concentration of cytokines was analyzed with ELISA kit (eBioscience, USA).

### Adhesion assay

THP-1 monocytes were labeled with BCECF/AM and resuspended in RPMI-1640 medium to a density of 1 × 10^6^ cells/ml. The resuspended cells were activated with Pam3CSK4 (1 μg/ml) or not, then incubated with PBS or various concentrations of Rn for 30 min at 37 °C. Cells were subjected to adhesion as previously described^9^.

### Migration assay

Costar Transwell (polycarbonate filter, 5  μm pore size) was coated with gelatin (2%) and dried for 2 h. THP-1 (5 × 10^5^ cells) incubated with Pam3CSK4 (1 μg/ml) or PBS was treated with various concentration of Rn at 37 °C for 30 min, and then cells were seeded onto the upper chamber. The bottom chamber was only added RPMI-1640 medium. Migration assay of THP-1 monocyte was measured as described before^37^.

### FITC-conjugated Rn binding assay

Human monocyte THP-1 stimulated with Pam3CSK4 (1 μg/ml) were incubated with FITC-conjugated Rn or FITC-conjugated BSA. Rn Binding assay was analyzed as previously described^9^.

### Western blotting of protein kinases

In the presence or absence of 1 μg/ml Pam3CSK4, THP-1 cells were treated with various concentration of Rn at 37 °C for 30 min. After incubation, cells were harvested and subjected to Western blotting assay as previously described^9^.

### Subcellular fractionation

Membrane and cytoplasmic protein were obtained with Proteo JET^TM^ membrane protein extraction kit (Thermo Fermentas, EU) according to manufacturer’s protocol.

### RNA interference

The small interfering RNAs (siRNAs) were transfected into THP-1 cells, including integrin αV, integrin αM, FAK, Akt and non-targeting siRNA (MDBio Inc., Taiwan). The siRNA sense sequences: human integrin αV, 5′-CACUCCAAGAACAUGACUA-3′ and 5′-GACUGAGCUAAUCUUGAGA-3′; human integrin αM, 5′-UUGAGGAGCAGUUUGUUUCCAAGGG-3′; FAK, 5′-GAGAAGGCUCAGCAAGAAG-3′; Akt, 5′-UCCUGGUUGUAGAAGGGCA-3′; non-targeting, 5′-UUCUCCGAACGUGUCACGU-3′.

### Animal model of cecal ligation and puncture (CLP)-induced sepsis

Male BALB/c mice with about 25 g were used in studies. Mice were maintained in accordance with the *Guide for the Care and Use of Laboratory Animals* (Institute of Laboratory Animal Resources, 1996) and were treated ethically. The mice were maintained on a 12-hr light/dark cycle under controlled temperature (20 ± 1 °C) and humidity (55 ± 5%). Animals were given continuous access to food and water. The protocol of animal study was approved by *Laboratory Animal Center, College of medicine, National Taiwan University*.

CLP-induced sepsis was induced with modification according to previously described method^38^. After mouse was anesthetized with pentobarbital (50 mg/kg, i.p.), cecum and adjoining intestine were taken out through a 20 mm incision. The cecum was tightly ligated with 3-0 silk suture 1 cm from the cecal tip and punctured through once with a 23-gauge needle. The cecum was then returned to the abdominal cavity and the laparotomy site was sutured. In sham controls, the cecum was taken out and returned without ligation or puncture.

### Mice whole blood and serum collection

Whole blood of mice was obtained from orbital venous sinus and collected at different time (6 h or 24 h after surgery). After centrifugating at 1000 × g for 10 min, plasma was separated and collected. The plasma concentration of cytokines, IL-6, TNF-α, IL-1β and MCP-1, were measured with ELISA kit following the manufacturer’s protocol (eBioscience, USA).

### Plasma glucose Measurement

Blood was obtained from eyes by puncture, and plasma glucose levels were measured at 6, 24 and 48 hr after sham or CLP with glucose liquicolor kit following the manufacturers protocol (Human, Germany).

### Measurement of vital signs using the tail-cuff method

A thermostatically regulated, heating platform was used to maintain body temperature at 37 °C. During the experiment, conventional non-invasive blood pressure and heart rate in conscious mice was measured using the tail-cuff method (BP-2000; Visitech Syatems, Inc., Apex, NC, USA), for the measurement of blood pressure.

### Histological examination

Lung, liver and kidney segments were fixed in 10% v/v phosphate-buffered formalin for 48 ~ 72 hr and then embedded in paraffin. Next, the samples were sectioned (5 μm) using a microtome, stained with H&E, and examined with light microscopy at ×200 magnifications.

### Statistical analysis

Data are presented as mean ± SEM. Analysis of two groups were assessed by unpaired Student’s *t* test. Three or more groups were compared by one-way ANOVA and Newman-Keuls multiple comparison test. P values smaller than 0.05 (*p* < 0.05) was considered as significant difference.

## Additional Information

**How to cite this article**: Hsu, C.-C. *et al*. Snake Venom Disintegrin Inhibits the Activation of Toll-Like Receptors and Alleviates Sepsis through Integrin alphaVbeta3 Blockade. *Sci. Rep*. **6**, 23387; doi: 10.1038/srep23387 (2016).
